# The role of gut microbiota dysbiosis in the inflammatory pathogenesis of diabetic retinopathy

**DOI:** 10.3389/fimmu.2025.1604315

**Published:** 2025-07-07

**Authors:** Liqing Xie, Wenjian Lin

**Affiliations:** Department of Endocrinology, First Affiliated Hospital of Harbin Medical University, Harbin, China

**Keywords:** diabetic retinopathy, inflammation, gut microbiota, gut metabolites, microglia

## Abstract

Diabetic retinopathy, a prevalent microvascular complication of diabetes mellitus, is characterized by its increasing global prevalence and stands as the leading cause of visual impairment and blindness in adults. The pathogenesis of diabetic retinopathy involves multifactorial interactions, among which inflammatory responses play a pivotal role in disease progression. With the emergence of the “gut-retinal axis” concept, growing evidence has elucidated the intricate association between gut microbiota dysbiosis and the development of diabetic retinopathy. Studies have revealed significant differences in gut microbiota composition and diversity between patients with diabetic retinopathy and those without diabetic retinopathy. Dysbiosis of the gut microbiota compromises intestinal barrier integrity, thereby facilitating the translocation of intestinal metabolites into systemic circulation. This process may trigger the activation of systemic inflammatory responses, thus contributing to the pathogenesis and progression of diabetic retinopathy. This review examines the metabolic disturbances and systemic inflammatory responses induced by gut microbiota dysbiosis in diabetes, providing an in-depth analysis of how gut microbiota dysbiosis influences the inflammatory mechanisms underlying diabetic retinopathy. Furthermore, it summarizes the protective effects of anti-diabetic drugs on diabetic retinopathy by modulating the intestinal microenvironment, offering novel perspectives for the treatment of diabetic retinopathy.

## 1 Diabetic retinopathy and gut microbiota

Diabetes mellitus, a chronic metabolic disorder of global importance, has seen a steady increase in prevalence. According to epidemiological data from the International Diabetes Federation (IDF), the global diabetic population was estimated at 537 million in 2021, with projections suggesting a rise to 783 million by 2045 (1). Diabetic retinopathy (DR), one of the most common microvascular complications of diabetes, has drawn significant public health attention due to its high incidence and potential to cause vision loss. Current epidemiological estimates indicate that the global burden of DR will grow substantially, from 103.12 million cases in 2020 to 160.5 million by 2045 (2). DR is the leading cause of vision impairment and the primary cause of blindness in adults, underscoring the critical importance of managing DR to prevent blindness and improve quality of life. The development of DR is complex, involving multiple factors such as microvascular changes, neurodegeneration, metabolic dysfunction, genetic predisposition, as well as immune and inflammatory mechanisms (3). The gut microbiota has emerged as a new area of focus in DR research and is gaining increasing attention. With the introduction of the “gut-retina axis” concept, the connection between gut microbiota and DR has been increasingly supported.

The gut microbiota refers to the complex community of microorganisms living in the human gastrointestinal tract, primarily consisting of bacteria, fungi, viruses, and other microbes. These microbial populations interact with the host through multiple mechanisms, significantly influencing human health. The intestinal microbiota is mainly composed of two major bacterial phyla: Firmicutes (Gram-positive), which include Clostridium, Enterococcus, Luminococcus, and Lactobacillus; and Bacteroidetes (Gram-negative), which include Bacteroides and Prevotella species. These microbial communities maintain a delicate symbiotic relationship with the host, playing critical roles in nutrient metabolism, immune regulation, and intestinal homeostasis (4). Research has shown that gut microbiota dysbiosis is strongly linked to various metabolic and systemic disorders, including hypertension (5), diabetes mellitus (6), cardiovascular diseases (7), and obesity (8). The pathogenic mechanisms involve the gut microbiota’s ability to directly modulate host metabolic signaling pathways and produce bioactive metabolites, thereby inducing chronic low-grade inflammation, triggering autoimmune responses, and disrupting metabolic homeostasis. These complex interactions ultimately lead to systemic microenvironmental changes that contribute to disease progression (1). Alterations in gut microbiota composition and metabolite profiles show strong association with DR progression. Research demonstrates that intermittent fasting significantly reshapes gut microbiota structure, prevents retinopathy in diabetic mouse models, and prolongs survival, indicating the gut microbiota’s involvement in DR pathophysiological processes (9). Gut microbiota dysbiosis induces both local and systemic inflammation, directly influencing diabetes development and its microvascular complications, including DR onset and progression. Therefore, a deeper understanding of the potential connections between gut microbiota and DR provides novel insights for clinical treatment of DR. This review focuses on DR inflammatory mechanisms, outlining systemic inflammatory responses and retinal neurovascular inflammatory damage triggered by gut microbiota alterations under diabetic conditions. Furthermore, it explores how various anti-diabetic medications may indirectly protect the retina by modulating gut microbiota structure and function, thereby controlling DR progression, reducing vision loss risk, and improving patient quality of life.

## 2 The inflammatory mechanisms of DR

DR is a chronic “metabolic-inflammatory” disorder in which retinal neurons, vascular cells, and glial cells collectively form the neurovascular unit. As DR progresses, neurovascular coupling becomes disrupted, and the blood-retinal barrier (BRB) is compromised. The resulting inflammatory microenvironment stems from complex interactions among immune cells, pro-inflammatory mediators, and vascular endothelial cells, ultimately driving microvascular dysfunction (10). Microglia, the retina’s resident innate immune cells, play a key role in both neurodegeneration and vascular damage in DR. Under normal physiological conditions, they remain in a quiescent state, continuously monitoring and maintaining retinal homeostasis. However, in response to metabolic stress, microglia become activated, undergoing morphological changes, migrating to injury sites, and releasing inflammatory mediators, thereby engaging in immune response mechanisms (11). Studies have shown that activated microglia exhibit two distinct polarization states: the pro-inflammatory (M1) phenotype and the anti-inflammatory (M2) phenotype. In early-stage DR, microglia primarily assume the M2 phenotype, which provides retinal protection by clearing cellular debris through phagocytosis and secreting neurotrophic factors, thereby promoting neuronal survival and tissue repair. However, in advanced disease stages, a phenotypic shift occurs with progressive predominance of the M1 phenotype, marked by increased production of cytotoxic mediators such as interleukin-1 (IL-1) and tumor necrosis factor-α (TNF-α), ultimately leading to retinal microvascular damage (12, 13). The reciprocal interactions between microglia and endothelial cells result in an upregulation of inflammatory factor secretion, consequently fostering the development of a pro-inflammatory microenvironment (10). These interconnected mechanisms work synergistically to drive DR pathogenesis.

## 3 Metabolic disorders and systemic inflammation caused by dysbiosis of gut microbiota in a high-glucose environment

### 3.1 Microbiota dysbiosis triggers inflammatory response

**Figure 1:**
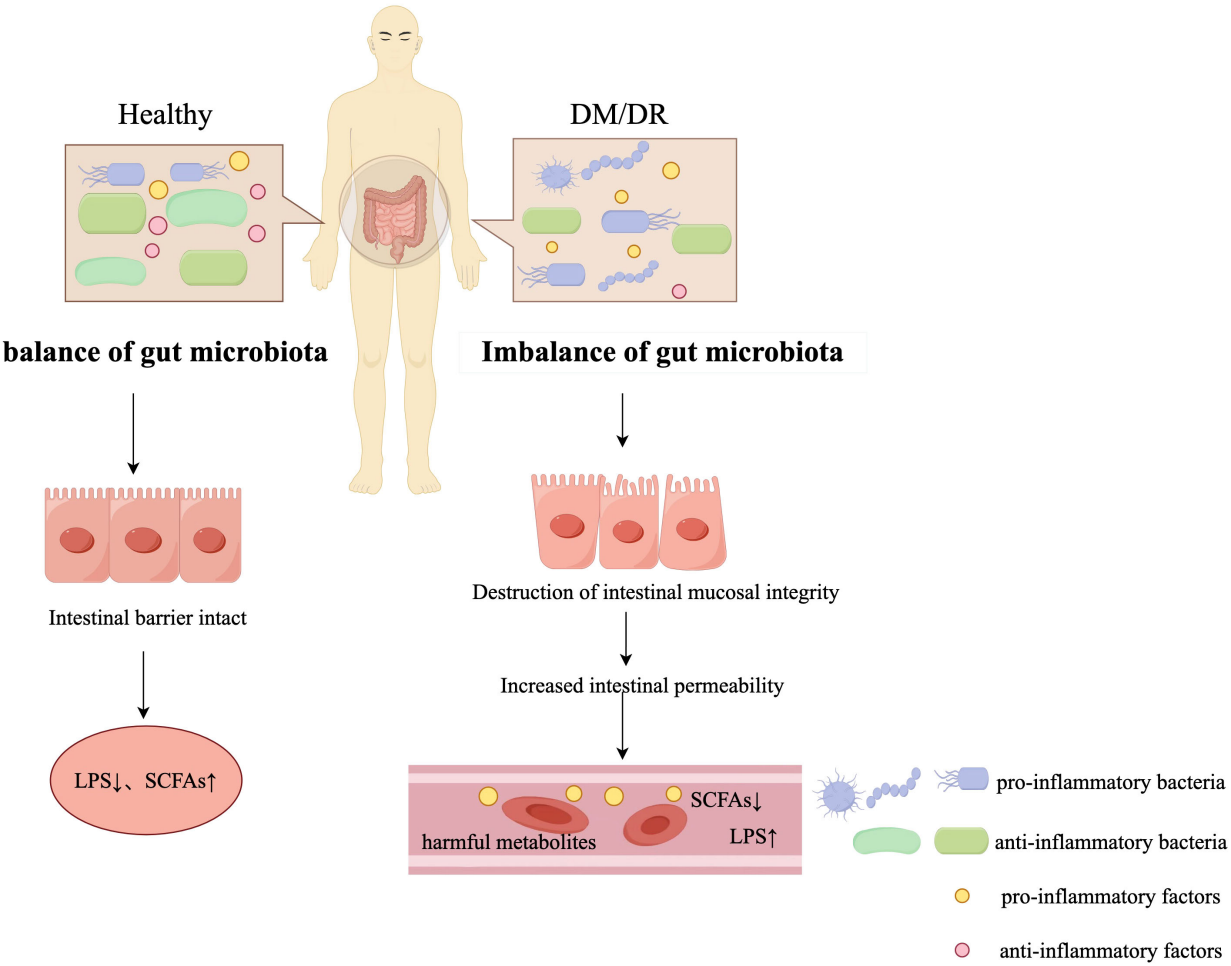
Differences in Gut Microbiota: A Comparison between Diabetic and Healthy Populations (by Figdraw). In the healthy state, the gut microbiota remains in balance and the anti-inflammatory bacteria predominate in the gut microbiota, helping to maintain the integrity of the intestinal barrier and the production of anti-inflammatory metabolites such as SCFAs. However, in the DM/DR state, the gut microbiota becomes imbalanced, with an increase in pro-inflammatory bacteria, leading to damage to the integrity of the gut mucosa and increased gut permeability. This imbalance leads to increased levels of pro-inflammatory factors, LPS, and decreased levels of SCFAs in the circulation, which will trigger systemic inflammation. In the legend, pro-inflammatory bacteria are shown in blue, anti-inflammatory bacteria in green, pro-inflammatory factors in yellow, and anti-inflammatory factors in pink.

In patients with diabetes mellitus, chronic hyperglycemia causes significant changes in the gut microenvironment, resulting in microbial dysbiosis and disruption of the homeostatic balance between anti-inflammatory and pro-inflammatory bacterial populations. This pathological cascade ultimately leads to systemic inflammatory responses through multiple pathways. Patients with T2DM show distinct gut microbiota composition compared to healthy controls(HC), with significantly reduced alpha diversity. T2DM patients have decreased abundance of anti-inflammatory bacteria including Roseburia, Lachnospira, Coprococcus, Phascolarctobacterium, and Anaerostipes, while demonstrating increased levels of the pro-inflammatory Methanobrevibacter (14). Elevated pro-inflammatory bacterial populations disrupt intestinal mucosal integrity, impairing gut barrier function. This impairment enables bacterial components and metabolites to enter circulation, inducing chronic systemic inflammation. The resulting inflammatory cascade directly contributes to the development of T2DM (15) (refer to **Figure 1**). Clinical studies have demonstrated that the pathogenesis of T2DM is closely linked to elevated serum levels of multiple proinflammatory cytokines and chemokines. In diabetic patients, peripheral blood levels of inflammatory markers such as ICAM-1, IL-6, IL-8, MCP-1, IL-1β, Vascular Cell Adhesion Molecule-1 (VCAM-1), and Vascular Endothelial Growth Factor (VEGF) are significantly higher than those in healthy individuals. These inflammatory factors circulate in the bloodstream and act on various tissues and organs, thereby triggering a systemic inflammatory response (16).

Within the gut microbiota’s complex regulation of inflammation, Roseburia species, especially Roseburia intestinalis, display multifaceted anti-inflammatory effects mediated primarily through their production of butyrate, a metabolite that effectively inhibits NF-κB signaling pathway activation. Furthermore, Roseburia intestinalis promotes the production of regulatory T cells (Tregs), upregulates transforming growth factor-β (TGF-β) expression, and enhances secretion of the anti-inflammatory cytokine IL-10. Moreover, it potently inhibits the secretion of pro-inflammatory cytokines, including interleukin-17 (IL-17) and interferon-gamma (IFN-γ), thereby exerting significant anti-inflammatory effects (17, 18). Additionally, Bacteroides fragilis, Akkermansia muciniphila, and Lactobacillus species (Lactobacillus plantarum and Lactobacillus casei) have been shown to markedly enhance the production of anti-inflammatory cytokines such as interleukin-10 (IL-10), collectively promoting intestinal immune homeostasis (18–22).

### 3.2 Metabolic disorders induced by gut microbiota dysbiosis

**Figure 2:**
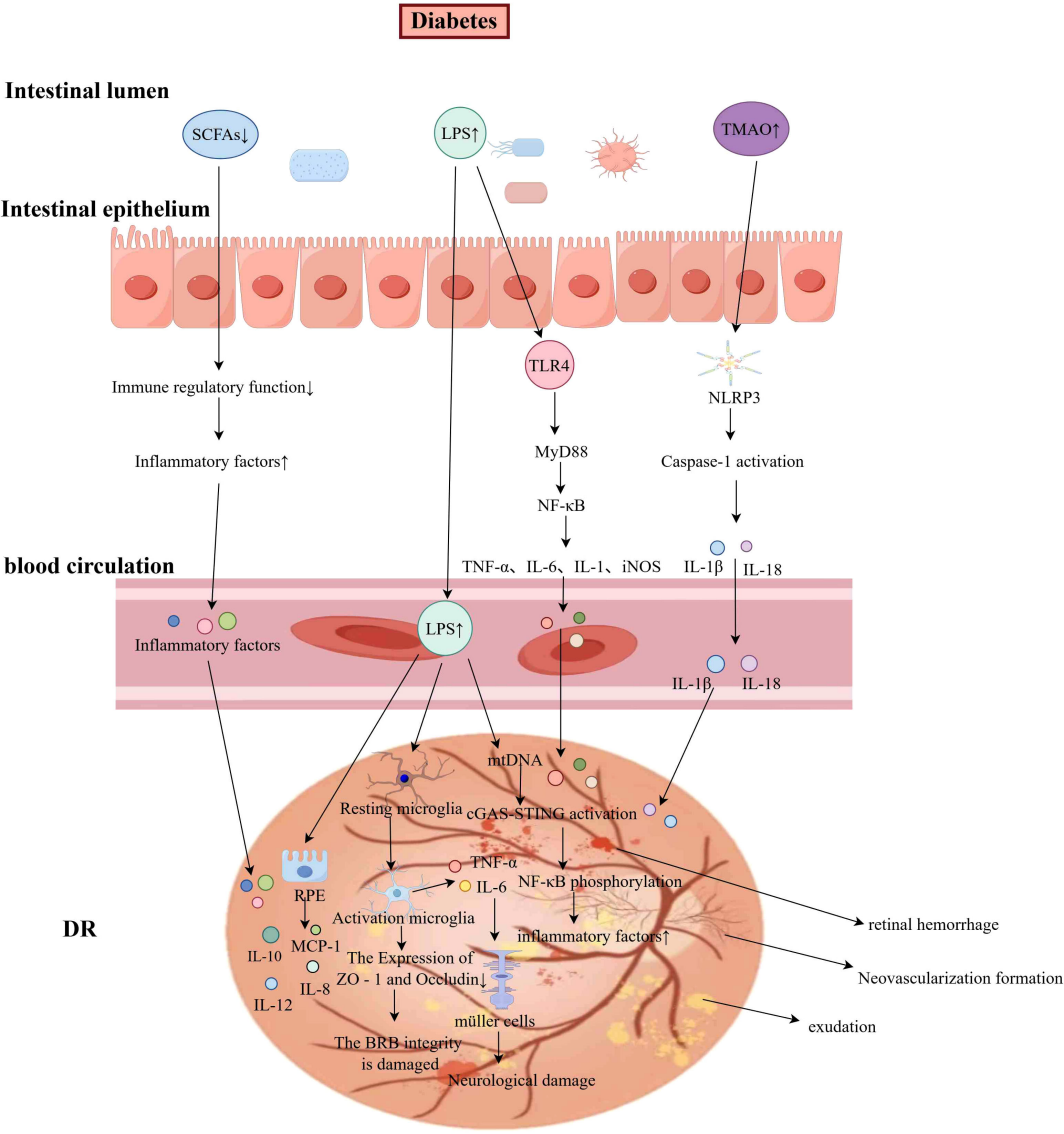
The Mechanism of Gut Microbiota in DR (by Figdraw). Under diabetic conditions, gut microbiota dysbiosis results in impaired intestinal barrier function and increased intestinal permeability, leading to reduced levels of SCFAs and elevated levels of TMAO and LPS. LPS activates the TLR4/MyD88/NF-κB signaling pathway in macrophages, promoting the release of pro-inflammatory cytokines such as TNF-α and IL-6. Additionally, LPS stimulates microglia and RPE cells, triggering inflammatory responses via the cGAS-STING pathway. These inflammatory mediators activate Müller cells, contributing to neuroretinal damage. The elevated TMAO levels activate the NLRP3 inflammasome, inducing the secretion of IL-1β and IL-18. The interplay of these inflammatory factors may ultimately result in retinal hemorrhage, exudation, and pathological neovascularization.

Lipopolysaccharide (LPS) is an endotoxin that is mainly found in the cell wall of Gram-negative bacteria. Studies have found that individuals with T2DM have higher levels of LPS in their bloodstream compared to HC. This phenomenon is associated with gut microbiota dysbiosis (23, 24). In the event of dysbiosis of the gut microbiota, certain intestinal bacteria (e.g. Desulfovibrionaceae) will affect the production of LPS (25). Toll-like Receptor 4 (TLR4) represents an essential receptor for recognizing LPS, which plays a critical role in the host’s immune response to bacterial infection (26). LPS activates the TLR4/Myeloid differentiation primary response protein 88(MyD88)/NF-κB signaling cascade in macrophages, triggering synthesis of pro-inflammatory mediators including TNF-α, IL-6, IL-1β, and inducible nitric oxide synthase (iNOS), which collectively drive the inflammatory response (refer to **Figure 2**). This inflammatory cascade activation results in the stimulation of serine kinases, specifically c-Jun N-terminal kinase (JNK) and IκB kinase (IKK). These kinases subsequently phosphorylate serine residues on Insulin Receptor Substrate (IRS), a critical component of the insulin receptor pathway. This post-translational modification impairs insulin signaling transduction, ultimately leading to the development of cellular insulin resistance (27). Furthermore, LPS has been demonstrated to activate the NOD-like receptor protein 3 (NLRP3) inflammasome complex, leading to the maturation and secretion of pro-inflammatory cytokines, particularly interleukin-1β (IL-1β) and interleukin-18 (IL-18). Releasing these cytokines will exacerbate insulin resistance, impair pancreatic β-cell function, and contribute to systemic inflammation (28, 29). Concurrently, LPS has been shown to induce structural and functional alterations in intestinal epithelial tight junctions by disrupting the organization and distribution of key tight junction proteins. The compromised barrier function facilitates the translocation of bacterial metabolites and endotoxins into the systemic circulation, thereby initiating a cascade of events that culminate in metabolic endotoxemia (30, 31) (refer to **Figure 1**).

Short-chain fatty acids (SCFAs) are metabolites generated by the gut microbiota during the fermentation of dietary fibers, including acetate, propionate, and butyrate. They regulate the host’s immune response, energy metabolism, and intestinal health (32). Zhao et al., through fecal SCFA analysis, demonstrated that SCFA levels were significantly lower in the T2DM group than in the healthy group (33). SCFAs exert anti-inflammatory properties through the suppression of pro-inflammatory mediators, including cytokines (TNF-α, IL-6, and IL-1β) as well as chemokines such as C-C motif ligand 2 (CCL2), C-C motif ligand 8 (CCL8), C-X-C motif ligand 5 (CXCL5), C-X-C motif ligand 8 (CXCL8), and C-X-C motif ligand 10 (CXCL10). These immunomodulatory effects are primarily mediated through the inhibition of the NF-κB p65 signaling pathway in immature histone H4 (H4) intestinal epithelial cells. The downregulation of this critical inflammatory pathway contributes to attenuating intestinal inflammation (34). In addition, Researchers have demonstrated that SCFAs regulate immune cell activity by activating G protein-coupled receptors (GPCRs), such as G Protein-Coupled Receptor 41 (GPR41) and G Protein-Coupled Receptor 43 (GPR43). These receptors suppress oxidative stress and abnormal activation of the NF-κB pathway, thereby inhibiting intestinal endotoxemia and inflammatory responses (35, 36). Diminished levels of SCFAs disrupt immune homeostasis, thereby enhancing the secretion of inflammatory cytokines and initiating a systemic inflammatory cascade (refer to **Figure 2**).

Bile Acids(BAs) play a crucial role in regulating the gut microbiota and host metabolism. Under healthy conditions, the synthesis, secretion, and reabsorption of BAs are closely associated with the composition and function of the gut microbiota, forming a dynamic balance between the two. However, in diabetes, gut microbiota dysbiosis disrupts the normal metabolic processes of BAs. Recent studies have revealed new roles of BAs in glucose metabolism (37), lipid metabolism (38),energy homeostasis (39), and systemic inflammatory responses. Studies have shown that in the fed state, the total BAs concentration is higher in patients with T2DM than in non-diabetic individuals (37). The study by Wang et al. demonstrated a significant upregulation of bacteria associated with BAs metabolism, such as Lactobacillus and Bifidobacterium, in patients with T2DM. These microbial populations are enriched with bile salt hydrolase (BSH), which plays a crucial role in BAs deconjugation. Multi-omics analysis further revealed that these bacteria are significantly associated with inflammatory genes, including MYD88 and NF-κB. Consistent with these findings, alterations in related inflammatory genes were also observed in the livers of T2DM mice, underscoring the correlation between BAs metabolism and inflammation (40). These results suggest that changes in the gut microbiota of diabetic patients may modulate inflammatory responses through their impact on bile acid metabolism.

In the diabetic state, dysbiosis of the gut microbiota and changes in its metabolites destroy the intestinal barrier function and increase intestinal permeability, leading to the release of bacterial metabolites (such as LPS) into the blood circulation, activating the immune system and triggering systemic inflammation. This inflammatory response is not only limited to the intestine, but also spreads to organs such as the liver, adipose tissue, and retina, affecting metabolic function and increasing the risk of DR and other related complications (41).

## 4 Hyperglycemia-induced gut microbiota dysbiosis drives retinal inflammatory microenvironment formation

### 4.1 Gut microbiota changes in DR patients

The gut microbiota composition demonstrated significant abundance variations between DR and T2DM patients. At the phylum level, DR patients exhibited reduced Firmicutes abundance with increased Bacteroidetes and Desulfobacterota levels compared to T2DM patients. At the family level, Pasteurellaceae abundance decreased in DR patients, while Eubacteriaceae showed an increasing trend (42). At the genus level, in DR patients, the abundance of anti-inflammatory bacteria such as Faecalibacterium, Bifidobacterium, Ruminococcus, Turicibacter, Streptococcus, Lactobacillus, and Butyricimonas was significantly reduced, while that of pro-inflammatory bacteria such as Shigella was significantly increased (14). Furthermore, Zhou et al. reported a significant increase in Prevotella abundance among DR patients (43). Studies have shown that Prevotella contributes to the pathogenesis of chronic inflammatory diseases through TLR2-mediated mechanisms. Prevotella activation of Toll-like receptor 2 (TLR2) stimulates antigen-presenting cells to produce IL-23 and IL-1, thereby promoting Th17 cell-mediated inflammatory responses (44). As the primary effector molecule of Th17 cells, interleukin-17A (IL-17A) contributes to retinal endothelial cell death through the IL-17A/IL-17 receptor (IL-17R) signaling axis. This pathway mediates its effects through sequential activation of the Act1 adaptor protein and Fas-associated death domain (FADD), ultimately triggering caspase-dependent apoptotic pathways in retinal vascular endothelial cells (45–47). These molecular mechanisms suggest that Prevotella enrichment in DR patients may exacerbate retinal tissue damage through Th17-mediated inflammatory processes.

### 4.2 Retinal injury mediated by inflammatory factor changes

Alterations in gut microbiota composition in diabetic patients have been implicated in the pathogenesis of systemic inflammation, which subsequently contributes to retinal damage in diabetic individuals. In a diabetic rat model, Di et al. demonstrated a significant correlation between increased abundance of Enterobacterial species and elevated serum levels of IL-6. Furthermore, Prevotellaceae species have been identified to show positive correlations with increased concentrations of IL-8, VEGF, and IL-1β. Similarly, Bacteroides species have been associated with upregulated levels of both IL-8 and IL-1β. Klebsiella species have been specifically linked to elevated VEGF levels in the systemic circulation. Faecalibaculum and Corynebacterium were respectively related to the decrease in the levels of inflammatory factors, IL-1β and MCP-1 (25). Moreover, experimental evidence indicates that diminished abundance of Bifidobacterium species is associated with upregulated expression of inflammatory mediators, including IL-6, TNF-α, and IFN-γ, while simultaneously leading to significant downregulation of the anti-inflammatory cytokine IL-10 (16, 48). Hyperglycemia induces a significant upregulation of inflammatory mediators, including pro-inflammatory cytokines (TNF-α, IL-1β, IL-6) and chemokines (MCP-1, IL-8). These molecular alterations activate critical signaling pathways like NF-κB and Janus kinase/signal transducer and activator of transcription (JAK/STAT) pathways, which subsequently trigger oxidative stress and initiate inflammatory cascades. The resulting inflammatory responses compromise BRB integrity, leading to increased vascular permeability and facilitating the transmigration of inflammatory mediators into retinal tissues. Concurrently, the upregulated pro-inflammatory factors stimulate vascular endothelial cells to secrete pro-angiogenic factors, thereby promoting pathological neovascularization. This persistent inflammatory-angiogenic interplay leads to progressive disruption of retinal architecture and function, ultimately contributing to the progression of DR (23, 49, 50).

### 4.3 Metabolites’ potential impact on the retina

Dysbiosis of the gut microbiota leads to impairment of intestinal barrier integrity, facilitating the translocation of intestinal metabolites including LPS, SCFAs, BAs, and trimethylamine N-oxide (TMAO) across the intestinal epithelium into systemic circulation. Following their systemic distribution, these microbial-derived metabolites may ultimately reach the retinal compartment, where they have the potential to modulate the functional activity of resident immune cells (particularly microglia and Müller cells) and vascular endothelial cells. This cascade of events subsequently induces localized inflammatory responses and contributes to the pathogenesis of retinal neurovascular damage.

#### 4.3.1 LPS

As a primary initiator of inflammatory responses, LPS activates multiple retinal cell populations, including microglia, Müller cells, retinal pigment epithelial (RPE) cells, and retinal microvascular endothelial cells (RMECs), thereby inducing the release of inflammatory mediators and playing a pivotal role in the pathogenesis of DR. LPS possesses the capacity to polarize microglia towards a pro-inflammatory (M1) phenotype, thereby enhancing their proliferation, migration, and phagocytic abilities. This conversion is accompanied by the secretion of pro-inflammatory cytokines, including IL-6 and TNF-α, which subsequently trigger retinal inflammatory cascades. Moreover, these cytokine mediators initiate the activation of retinal glial cells, particularly Müller cells, ultimately leading to neuroinflammatory injury within the retinal tissue (51–53). LPS-activated microglia modulate the tight junction integrity of endothelial cells through downregulation of tight junction proteins, including ZO-1 and occludin, as well as by altering the expression patterns of endothelial surface markers CD31 and CD34. This microglial-mediated disruption of endothelial barrier function compromises vascular integrity, leading to breakdown of the BRB and facilitating pathological neovascularization (54). The RPE, which constitutes the outer component of the BRB, undergoes a phenotypic transition to a pro-inflammatory state upon LPS exposure. In this activated state, RPE cells secrete a spectrum of inflammatory mediators, including interleukin-4 (IL-4), IL-6, IL-8, IL-10, IL-17, IFN-γ, MCP-1, and VEGF. These inflammatory mediators induce structural alterations in tight junction complexes, ultimately resulting in RPE degeneration and progressive disruption of the outer BRB (55). Research has demonstrated that under pathological stimuli such as LPS and high glucose conditions, mitochondrial DNA (mtDNA) is released from mitochondria into the cytoplasm of RMECs. This cytoplasmic mtDNA is subsequently recognized by the DNA sensor cyclic GMP-AMP synthase (cGAS), leading to the activation of the STING-TBK1 signaling pathway and subsequent upregulation of inflammatory cytokine expression. The enhanced secretion of these inflammatory cytokines promotes the adhesion of inflammatory cells to vascular endothelial cells, ultimately resulting in the disruption of BRB function (56, 57) (refer to **Figure 2**). Liu et al. discovered that retinal endothelial cells demonstrate significantly elevated expression of STING protein, particularly within the preretinal and intraretinal neovascular membranes, as well as in the surrounding microglial cells. This observation suggests that STING pathway activation plays a crucial role in mediating both neovascularization and inflammatory responses in DR. Furthermore, studies utilizing STING-knockout mice have demonstrated that the absence of STING signaling attenuates endothelial cell senescence, inflammatory activation, and capillary degeneration. These findings provide compelling evidence that the STING pathway contributes to the pathogenesis of DR through its promotion of inflammatory cascades and senescence-related processes in endothelial cells (58, 59) (refer to **Figure 2**).

#### 4.3.2 SCFAs

SCFAs exert beneficial effects on DR through their anti-inflammatory properties, immunomodulatory functions, and protective effects on vascular endothelial cells. Studies have revealed that SCFAs are capable of traversing the blood-eye barrier via systemic circulation, subsequently reducing intraocular inflammation and modulating cellular functions (60). Huang et al. demonstrated that butyrate supplementation significantly reduces blood glucose levels in diabetic mice and decreases their food and water intake. Furthermore, butyrate has been shown to ameliorate retinal thinning in diabetic mice, suppress the activation of retinal microglia, and improve physiological functional parameters in electroretinography (61). Concurrently, SCFAs have been found to inhibit the production of pro-inflammatory cytokines, including TNF-α, C - X - C motif chemokine 12 (CXCL 12), and C - X - C motif chemokine 1 (CXCL1), in LPS-stimulated retinal astrocytes (RACs). Additionally, SCFAs are capable of reducing LPS-induced immune cell infiltration (including lymphocytes and monocytes) and inhibit leukocyte migration (60). These findings suggest that butyrate may possess therapeutic potential for delaying the progression of DR.

#### 4.3.3 BAs

BAs play a crucial protective role in DR by regulating inflammatory responses and inhibiting the activation of inflammatory signaling pathways. In a db/db mouse model, Beli et al. demonstrated that intermittent fasting induces alterations in gut microbiota composition, resulting in enhanced production of the secondary bile acid tauroursodeoxycholic acid (TUDCA), which exerts protective effects on the retina. The elevated systemic levels of TUDCA are capable of crossing the BRB through circulatory transport. The bile acid G protein-coupled receptor 5 (TGR5), the primary receptor for TUDCA, is predominantly expressed in the retinal ganglion cell layer. TUDCA-mediated activation of TGR5 signaling significantly downregulates the expression of the pro-inflammatory cytokine TNF-α in mouse models, suggesting a potential therapeutic strategy for delaying DR progression (9, 62). In Müller cells, TGR5 activation mitigates mitochondrial calcium overload and dysfunction through the inhibition of endoplasmic reticulum (ER)-mitochondrial coupling-mediated Ca^2+^ transfer from the ER to mitochondria, consequently suppressing the cGAS/STING pathway-mediated inflammatory response (63). Additionally, Ouyang et al. demonstrated that ursodeoxycholic acid (UDCA) exerts a protective effect by inhibiting the activation of the NF-κB signaling pathway in the retinas of diabetic mice. This protective mechanism is achieved through the downregulation of inflammatory mediators, including TNF-α, IL-1, IL-6, ICAM-1, iNOS, and VEGF, as well as the restoration of tight junction proteins (claudin-1 and claudin-19) expression, thereby preserving BRB integrity and attenuating retinal inflammation (64).

#### 4.3.4 TMAO

Dysbiosis of the gut microbiota leads to the overexpression of TMAO, which exacerbates the pathological progression of DR through mechanisms such as promoting neovascularization and inducing inflammatory responses. Xue et al. identified elevated levels of TMAO in the serum and aqueous humor of patients with PDR. Further investigations revealed that TMAO enhances the proliferation, migration, and angiogenic capacity of human retinal microvascular endothelial cells (HRMECs) under high-glucose conditions, thereby accelerating the formation of neovascularization. Furthermore, TMAO exacerbates high glucose-induced degradation of the tight junction protein ZO-1 in HRMECs, potentially leading to increased vascular permeability. Concurrently, TMAO not only increases high glucose-induced reactive oxygen species (ROS) production but also promotes the activation of the NLRP3 inflammasome, thereby playing a significant role in driving inflammatory responses (65–67). Comparative studies have demonstrated that both the gene expression levels and protein content of NLRP3 in peripheral blood mononuclear cells (PBMCs) are significantly elevated in patients with DR compared to HC (30). Upon activation, the NLRP3 inflammasome recruits and activates downstream adaptor proteins, such as apoptosis-associated speck-like protein containing a caspase recruitment domain (ASC), which subsequently activates caspase-1. This cascade promotes the release and maturation of the pro-inflammatory cytokines IL-1β and IL-18, which are essential drivers of the inflammatory response (68–70) (refer to **Figure 2**).

## 5 Anti-diabetic drugs modulating gut microbiota: a new therapeutic approach for DR

The mechanisms of action of anti-diabetic drugs are not limited to their direct glucose-lowering effects. Emerging evidence indicates that these agents modulate the composition and functionality of the gut microbiota, thereby indirectly regulating the production of inflammatory mediators and the activation of inflammatory signaling pathways, which collectively contribute to their protective effects against DR (refer to **Table 1**). Consequently, a deeper understanding of the therapeutic potential of anti-diabetic drugs in modulating gut microbiota and suppressing inflammation may offer significant advantages for the clinical management of DR.

**Table 1:** Effects of anti-diabetic drugs on gut microbiota composition and metabolites.

Anti-Diabetic Drug		Increased gut bacteria	Decreased gut bacteria	Gut Metabolites	References
Biguanides	Metformin	Akkermansia muciniphila,Butyrivibrio, Bifidobacterium bifidum, Megasphaera, an operational taxonomic unit of Prevotella	Clostridiaceae02d06,Oscillospira,Barnesiellaceae	elevated levels of SCFAs	(71)
		Bifidobacterium,Escherichia,Akkermansia muciniphila	Intestinibacter	Increased SCFA and BA concentrations	(72)
GLP-1 receptor agonists	Liraglutide	Bacteroides, Lachnospiraceae, probiotic bacteria, Bifidobacterium	Prevotella species,Mollicutes	Increased levels of SCFAs	(73)
	Semaglutide	Alloprevotella, Alistipes	Ligilactobacillus, Lactobacillus	Increased levels of SCFAs	(74)
SGLT2 inhibitors	Dapagliflozin	Akkermansia muciniphila	Oscillospira	Reduced succinic acid levels and increased SCFA levels	(75)
a-glucosidase inhibitors	Acarbose	Bifidobacterium longum,Enterococcus faecalis	Enterobacteriaceae	Decreased LPS levels and increased SCFA levels	(76)
Traditional Chinese Medicine	SZ - A	Bacteroidaceae, Erysipelotrichaceae, Verrucomicrobia, Bacteroides, Faecalibaculum, Allobaculum	Rikenellaceae,Desulfovibrionaceae, Aerococcaceae, Alistipes, Desulfovibrio, Aerococcus	Decreased LPS levels and increased SCFA levels	(25)
	APS	Lactobacillus,Bifidobacterium,Muribaculum,Faecalibaculum, Lactobacillus	Enterobacteriaceae,Escherichia-Shigella,Parabacteroides	Increased SCFA levels	(77)
	LTF	Faecalibaculum,Corynebacterium,Firmicutes,Candi-datus_Saccharimonas, Romboutsia, Enterorhabdus	Bacteroidetes	Not determined.	(16)
	CQLT	Akkermansia, Clostridium_sensu_stricto_1	Blautia, Butyricimonas, Family_XIII_AD3011_group, Negativibacillus	Reduced levels of L-glutamine,fructose-6-phosphate, and asparagine	(78)

### 5.1 Biguanides

Metformin exerts regulatory effects on the gut microbiota and metabolic products (79, 80). Studies by Cuesta-Zuluaga et al. revealed that in patients with T2DM receiving metformin treatment, the abundance of beneficial bacteria, such as Akkermansia muciniphila, as well as specific microbial populations capable of producing SCFAs, including Butyrivibrio, Bifidobacterium bifidum, Megasphaera, and Prevotella, was increased. the abundance of potentially pathogenic microbial taxa, such as Clostridiaceae 02d06, Oscillospira, and Barnesiellaceae, was reduced (71). Similarly, Wu et al. demonstrated that metformin treatment in T2DM patients led to an increased abundance of Escherichia and Bifidobacterium within the gut microbiota, while the abundance of Intestinibacter was decreased. Furthermore, metformin treatment not only significantly elevated the fecal concentrations of SCFAs, such as propionate and butyrate, but also affected the metabolism of BAs, thereby leading to an increase in the concentration of BAs in the blood (72). In conclusion, these findings suggest that metformin may exert its anti-inflammatory effects by promoting the production of these metabolites, thereby mitigating the pathological progression of DR and providing protective benefits to the retina.

### 5.2 GLP-1 receptor agonists

GLP-1 is a peptide hormone secreted by intestinal L cells (81). It not only plays a role in regulating blood glucose levels but also modulates the composition and structure of the gut microbiota through activation of intestinal GLP-1 receptors (82). Liraglutide, a GLP-1 receptor agonist, has been shown to regulate the composition and function of the gut microbiota, reducing the Firmicutes-to-Bacteroidetes ratio. During treatment, liraglutide increases the abundance of beneficial bacteria, such as Bacteroides, Lachnospiraceae, probiotic species, and Bifidobacterium, which are capable of producing SCFAs. Concurrently, liraglutide reduces the relative abundance of opportunistic pathogens, including Prevotella species and Mollicutes. In addition, Liraglutide reduces circulating levels of insulin and IL-6 (73). Studies have revealed that semaglutide modulates the gut microbiota. In a db/db diabetic mouse model, treatment with semaglutide significantly enhanced the alpha diversity of the gut microbiota, with increased abundance of Alloprevotella and Alistipes, and decreased abundance of Ligilactobacillus and Lactobacillus. The elevated abundance of Alloprevotella and Alistipes is associated with enhanced production of SCFAs (74). Therefore, GLP-1 receptor agonists may exert protective effects against DR by modulating the composition of the gut microbiota and reducing the production of inflammatory mediators.

### 5.3 Sodium-glucose cotransporter 2 inhibitors

Dapagliflozin, a sodium-glucose cotransporter 2 (SGLT2) inhibitor, primarily lowers blood glucose levels by inhibiting SGLT2 in the kidneys, thereby reducing glucose reabsorption and promoting urinary glucose excretion. Beyond their glucose-lowering effects, SGLT2 inhibitors modulate the gut microbiota and their metabolic products, exerting beneficial effects on the host. Studies have demonstrated that treatment with dapagliflozin in diabetic mice leads to significant alterations in the gut microbiota, characterized by a reduced Firmicutes-to-Bacteroidetes ratio, a decreased abundance of Oscillospira, and an increased population of Akkermansia muciniphila (83). Studies have revealed that SGLT2 inhibitors reduce levels of the intermediate metabolite succinate while increasing butyrate levels in SCFAs (75). Succinate, by binding to its specific receptor GPR91, activates VEGF in retinal ganglion cells under hypoxic conditions, thereby promoting neovascularization (84). Therefore, SGLT2 inhibitors may exert protective effects against DR by modulating the gut microbiota, reducing succinate levels, and elevating butyrate levels.

### 5.4 Alpha-glucosidase inhibitors

Alpha-glucosidase inhibitors (α-GIs) primarily target the brush border of the upper small intestine, slowing carbohydrate catabolism and absorption through the inhibition of α-glucosidase activity (85). Studies have demonstrated that α-GIs not only directly regulate blood glucose levels but also confer indirect protective effects against DR by modulating the composition of the gut microbiota. Acarbose, a representative α-GI, has been shown to increase the abundance of culturable Bifidobacterium longum and Enterococcus faecalis, reduce the abundance of Enterobacteriaceae, and lower LPS levels in patients with T2DM following treatment (76). Acarbose administration induced alterations in the composition of the gut microbial community and the concentrations of SCFAs in mice. These modifications in the gut microbiota, coupled with the elevated levels of fecal SCFAs, collectively contributed to the extension of the mice’s lifespan (63). Consequently, α-GIs may exert a protective effect against DR through their regulatory influence on both the gut microbiota and gut metabolites.

### 5.5 Traditional Chinese medicine

In the field of exploring the treatment of DR, traditional Chinese medicine (TCM) has garnered increasing attention owing to its therapeutic advantages of multi-pathway and multi-target mechanisms. TCM formulations such as Sangzhi Alkaloid Tablets (SZ-A), Astragalus Polysaccharide (APS), Luotong Formula (LTF), and Compound Qilian Tablets (CQLT) have demonstrated potential therapeutic value for DR through their actions in modulating gut microbiota, reducing inflammatory mediators, and inhibiting inflammatory signaling pathways.

SZ - A, extracted from the traditional Chinese herbal medicine Morus alba L, has been proven to improve hyperglycemia in T2DM and has been approved for clinical diabetes treatment (86). Studies have shown that administration of SZ-A in diabetic KKAy mouse models enhances insulin sensitivity and stimulates the secretion of GLP-1 (25, 86). Additionally, SZ-A modulates the gut microbiota by increasing the relative abundance of beneficial microbial taxa, including Bacteroidaceae, Erysipelotrichaceae, Verrucomicrobia, Bacteroides, Faecalibaculum, and Allobaculum. However, it effectively reduces the proliferation of potentially pathogenic microorganisms, such as Rikenellaceae, Desulfovibrionaceae, Aerococcaceae, Alistipes, Desulfovibrio, and Aerococcus. Furthermore, SZ-A increases the concentrations of acetate and propionate in fecal matter while reducing serum endotoxin levels. It also downregulates the expression of multiple inflammatory mediators and chemokines, including IL-1β, IL-6, CCL4, and CCL5. Moreover, SZ-A inhibits the activation of the NF-κB signaling pathway, thereby attenuating inflammatory responses (25). Consequently, through its regulatory effects on gut microbiota composition and intestinal metabolites, coupled with its anti-inflammatory properties, SZ-A may exert an indirect beneficial impact on DR.

APS, a bioactive compound extracted from Astragalus membranaceus, exhibits significant regulatory effects on gut microbiota composition (87). Studies by Zhang et al. have demonstrated that APS treatment enhances the abundance of beneficial bacterial taxa, including Lactobacillus and Bifidobacterium, while reducing the levels of potentially pathogenic bacteria such as Enterobacteriaceae, Escherichia-Shigella, and Parabacteroides (77). Furthermore, APS has been shown to downregulate the expression of pro-inflammatory mediators, including TNF-α and IL-6 in serum (88). These cytokines play crucial roles in the pathogenesis of chronic inflammation and the progression of diabetes mellitus (87). In addition, APS enhances the abundance of beneficial bacterial taxa, including Enterobacteriaceae, Enterobacter, and Lactobacillus, thereby elevating the concentrations of SCFAs and further improving the intestinal microenvironment (77, 87). Further investigations by Liu et al. revealed that APS downregulates the expression of Kir2.1 protein in retinal Müller cells and suppresses the production of VEGF in these cells, thereby contributing to the prevention and treatment of DR (89). Consequently, APS may exert its protective effects against DR through multiple mechanisms, including modulation of gut microbiota, preservation of intestinal barrier integrity, inhibition of inflammatory responses, and direct actions on retinal Müller cells.

LTF, a traditional Chinese herbal formulation, is composed of Radix Astragali (Huangqi), Radix Salviae Miltiorrhizae (Danshen), Radix Notoginseng (Sanqi), Hirudo (Shuizhi powder), and Rhubarb (Dahuang). It is traditionally recognized for its properties of promoting blood circulation, resolving blood stasis, and unblocking meridians (90). Research has revealed that its therapeutic mechanisms extend beyond these traditional effects to include modulation of gut microbiota. Studies by Di et al. demonstrated that LTF not only exhibits hypoglycemic effects but also alters the gut microbiota composition in diabetic rats. Following LTF treatment, the α-diversity of the gut microbiota was markedly enhanced. Additionally, LTF increased the relative abundance of Firmicutes while reducing that of Bacteroidetes, resulting in an elevated Firmicutes-to-Bacteroidetes ratio. LTF treatment enhances the relative abundance of beneficial bacterial taxa, including Faecalibaculum and Corynebacterium, while reducing serum levels of pro-inflammatory mediators such as ICAM - 1, IL - 6, IL - 8, MCP - 1, VCAM - 1, VEGF, and IL - 1β. Furthermore, LTF attenuates retinal inflammation through inhibition of the NF-κB signaling pathway. Moreover, LTF administration reduces BRB permeability, increases retinal thickness, and ameliorates retinal microvascular damage (16). These findings suggest that LTF may exert preventive and therapeutic effects on the progression of DR through its modulation of gut microbiota and associated anti-inflammatory mechanisms.

CQLT, a traditional Chinese herbal formulation, is composed of Radix Astragali, Rehmannia glutinosa, Coptis chinensis, and Panax notoginseng. It is clinically used to treat symptoms associated with DR, including blurred vision, visual field loss, and vitreous hemorrhage. Recent advances in the understanding of DR pathogenesis have highlighted the pivotal role of gut microbiota in disease progression, with the therapeutic efficacy of CQLT being closely linked to its modulatory effects on gut microbiota. Studies by Jia et al. have demonstrated that CQLT modulates the abundance and diversity of gut microbiota, enhancing the relative abundance of beneficial bacterial taxa such as Akkermansia and Clostridium_sensu_stricto_1, while reducing the relative abundance of potentially pathogenic bacteria, including Blautia, Butyricimonas, Family_XIII_AD3011_group, and Negativibacillus, thereby improving gut microbiota composition. Furthermore, CQLT enhances intestinal metabolic function by regulating the levels of key metabolites, including L-glutamine, fructose-6-phosphate, and asparagine. The alterations in these metabolites are closely associated with critical metabolic pathways, including glycolysis/gluconeogenesis, alanine, aspartate and glutamate metabolism, and starch and sucrose metabolism, all of which play essential roles in maintaining retinal homeostasis. Additionally, CQLT inhibits retinal neuroinflammation by downregulating the expression of retinal nerve damage markers, such as glial fibrillary acidic protein (GFAP) and ionized calcium-binding adapter molecule 1 (Iba-1) (78). These findings suggest that CQLT may confer protective effects on retinal neurons in DR rats through its ability to modulate and improve gut microbiota composition.

## 6 Conclusions and prospects

Gut microbiota dysbiosis induces metabolic disorders and systemic inflammatory responses, playing an important role in the inflammatory pathogenesis of DR. Research has shown that DR patients exhibit significant alterations in the composition and diversity of their gut microbiota, which not only compromise intestinal barrier integrity but also modulate the expression of inflammatory mediators. Furthermore, changes in gut microbiota regulate the activity of inflammatory factors through the production of gut metabolites, including LPS, SCFAs, BAs, and TMAO. These inflammatory factors and metabolites reach the retina via the systemic circulation, thereby influencing the neurovascular inflammatory response in DR. Together, these pathological changes contribute to DR onset and progression. However, current research on gut microbiota dysbiosis is still in an early stage, and the studies used in this review may possess certain biases.

Although emerging evidence has begun to elucidate the impact of gut microbiota and metabolite alterations on DR, numerous aspects of this field warrant further investigation and clarification. The composition of the gut microbiota varies considerably due to factors such as diet, genetic predisposition, and environmental exposures, resulting in significant heterogeneity that complicates comparisons across distinct patient groups, particularly given the diverse medication regimens being used. Additionally, gut microbiota dysbiosis may play a stage-specific role in the progression of DR, but its precise molecular mechanisms and regulatory networks require further in-depth exploration.

Emerging evidence highlights several potential intervention strategies, including dietary and lifestyle modifications, supplementation with SCFAs and BAs derivatives, probiotic interventions, fecal microbiota transplantation (FMT), and engineered microbial therapies. Nevertheless, the safety and efficacy of most these approaches await further validation through large-scale clinical trials. Importantly, given the marked interindividual heterogeneity in gut microbiota profiles, personalized therapeutic strategies are expected to emerge as a pivotal direction for optimizing treatment efficacy in the future. Meanwhile, in-depth research on the interactions between anti-diabetic drugs and the gut microbiota has provided a more comprehensive understanding of their mechanisms of action. Beyond classical hypoglycemic effects, these drugs modulate gut microbiota composition, influence metabolite production, and attenuate inflammatory responses, collectively contributing to protective effects against DR. However, most current evidences are derived from animal models, and further human studies are needed to solidify these findings due to numerous uncontrolled variables. Thus, conclusions should be interpreted cautiously. Future studies should explore this field to elucidate the underlying mechanisms and develop novel therapies for DR.

## Glossary

DR: diabetic retinopathy
NVU: neurovascular unit
BRB: blood-retinal barrier
IL-1: interleukin-1
TNF-α: tumor necrosis factor-alpha
HC: healthy controls
T2DM: type 2 diabetes mellitus
STING: stimulator of interferon genes
IL-10: interleukin-10
IL-22: interleukin-22
Tregs: regulatory T cells
TGF-β: transforming growth factor-beta
IL-1β: interleukin-1 beta
IL-6: interleukin-6
IL-8: interleukin-8
IL-17: interleukin-17
IFN-γ: interferon-gamma
MCP-1: monocyte chemoattractant protein-1
ICAM-1: intercellular adhesion molecule-1
CD36: cluster of differentiation 36
VCAM-1: vascular cell adhesion molecule-1
VEGF: vascular endothelial growth factor
LPS: lipopolysaccharide
NLRP3: nod-like receptor protein 3
IL-18: interleukin-18
TLR4: toll-like receptor 4
MyD88: myeloid differentiation primary response protein 88
iNOS: inducible nitric oxide synthase
JNK: c-jun n-terminal kinase
IKK: ikB kinase
IRS: insulin receptor substrate
SCFAs: short-chain fatty acids
CCL 2: chemokine (C-C motif) ligand 2
CCL 8: chemokine (C-C motif) ligand 8
CXCL 5: chemokine (C-X-C motif) ligand 5
CXCL 8: chemokine (C-X-C motif) ligand 8
CXCL 10: chemokine (C-X-C motif) ligand 10
H4: histone H4
GPCRs: G protein-coupled receptors
GPR41: G protein-coupled receptor 41
GPR43: G protein-coupled receptor 43
BSH: bile salt hydrolases
TLR2: toll - like receptor 2
Th17: th17 - polarizing cytokines
IL - 17: interleukin - 17
IL - 23: interleukin - 23
IL - 17A: interleukin - 17A
IL - 17R: interleukin - 17 receptor
FADD: fas - associated death domain
JAK/STAT: Janus Kinase/Signal Transducer and Activator of Transcription
RPE: retinal pigment epithelium
IL-4: interleukin-4
mtDNA: mitochondrial DNA
RMECs: retinal microvascular endothelial cells
cGAS: cyclic GMP-AMP synthase
CXCL 12: C - X - C motif chemokine 12
CXCL1: C - X - C motif chemokine 1
RACs: retinal astrocytes
TUDCA: tauroursodeoxycholic acid
TGR5: G protein-coupled receptor 5
BAs: bile acids
TMAO: trimethylamine N-oxide
HRMECs: human retinal microvascular endothelial cells
ROS: reactive oxygen species
PBMCs: peripheral blood mononuclear cells
ASC: caspase recruitment domain
SGLT2: sodium-glucose cotransporter 2
α- GI: alpha - glucosidase
TCM: traditional Chinese medicine
SZ - A: sangzhi total alkaloids tablets
LTF: luo tong fang
CQLT: compound qilian tablets
APS: astragalus polysaccharide
Iba - 1: ionized calcium - binding adaptor molecule 1
FMT: fecal microbiota transplantation
